# Supplementary material for: Myeloid‐resident neuropilin‐1 promotes choroidal neovascularization while mitigating inflammation
Source: EMBO Mol Med. 2021 Apr 19;13(5):e11754. doi: 10.15252/emmm.201911754 (PMC8103107; doi:10.15252/emmm.201911754)
Supplement: Supplementary file 1 — Appendix [file EMMM-13-e11754-s003.pdf]

Title: Myeloid-resident Neuropilin-1 promotes Choroidal Neovascularization while Mitigating Inflammation

Authors:
Elisabeth MMA Andriessen[1#], François Binet[2,3#], Frédérik Fournier[4], Masayuki Hata[4], Agnieszka Dejda[3], Gaëlle Mawambo[4], Sergio Crespo-Garcia[3,4], Frédérique Pilon[3], Manuel Buscarlet[4], Karine Beauchemin[2], Veronique Bougie[2], Garth Cumberlidge[2], Ariel M Wilson[3], Steve Bourgault[6], Flavio A Rezende[3], Normand Beaulieu[2], Jean-Sébastien Delisle[5], Przemyslaw Sapieha[1,2,3,4*]

Affiliations:
Departments of [1]Biomedical Sciences, [3]Ophthalmology, [4]Biochemistry and Molecular Medicine and [5]Medicine, Maisonneuve-Rosemont Hospital Research Centre, University of Montreal, Montreal, Quebec, H1T 2M4, Canada.
[2]SemaThera Inc. 4 Westmount Square, Suite 160, Montreal, Quebec, H3Z 2S6, Canada.
[6]Department of Chemistry, Université du Québec à Montréal, Montreal, Quebec, H2L 2C4, Canada
# These authors contributed equally.

Contact: Przemyslaw (Mike) Sapieha, Ph.D.
Maisonneuve-Rosemont Hospital Research Centre
Research Centre 5415 Assomption Boulevard, Montreal,
QC, H1T 2M4, Canada.
TEL: (514) 252-3400 ext. 7711. FAX: (514) 252-3430
E-mail: mike.sapieha@umontreal.ca

Appendix Table S1

Graph	Data	P-value	Test used	Summary
**Figure 1**				
C	ELISA	0.0007	T-test	***
D	ELISA	0.0007	T-test	***
I	qPCR	0.0034	ANOVA + Dunnett's	**
J	qPCR	0.0361	ANOVA + Dunnett's	*
K	qPCR	0.0338, 0.0167	ANOVA + Dunnett's	*,*
M	qPCR	0.0189	ANOVA + Dunnett's	*
**Figure 2**				
B	FACS	0.0303	T-test	*
C	FACS	0.0301	T-test	*
D	qPCR	0.0001	T-test	***
E	WB	< 0.0001	T-test	****
L	CNV	0.0089	T-test	**
N	CNV	0.0069	T-test	**
**Figure 3**				
A	qPCR	0.0463	T-test	*
B	qPCR	0.0063	T-test	**
E	qPCR	0.0409	T-test	*
J	WB	0.0416	T-test	*
K	qPCR	0.0335	T-test	*
L	qPCR	0.0332	T-test	*
N	FACS	0.0028	T-test	**
O	FACS	0.0025	T-test	**
**Figure 4**				
F	CNV	0.0074	T-test	**
H	CNV	0.0005	T-test	***
J	CNV	< 0.0001, 0.0197	ANOVA + Tukey's	****,*
L	CNV	< 0.0001, 0.0238	ANOVA + Tukey's	****,*
**Expanded View Figure 1**				
D	FACS	0.0002	T-test	***
E	FACS	0.0075	T-test	**
**Expanded View Figure 3**				
A	qPCR	0.041	T-test	*

T-test = Student's unpaired t-test, ANOVA + Dunnett's = Ordinary one-way ANOVA with Dunnett's multiple comparisons test, ANOVA + Tukey's = Ordinary one-way ANOVA with Tukey's multiple comparisons test

Appendix Table S1: Table of p values
