# Supplementary material for: Myeloid‐resident neuropilin‐1 promotes choroidal neovascularization while mitigating inflammation
Source: EMBO Mol Med. 2021 Apr 19;13(5):e11754. doi: 10.15252/emmm.201911754 (PMC8103107; doi:10.15252/emmm.201911754)
Supplement: Supplementary file 2 — Expanded View Figures PDF [file EMMM-13-e11754-s001.pdf]

Expanded View Figures

Figure EV1. Mononuclear phagocyte-resident NRP1 expression in LysM-Cre/*Nrp1*$^{fl/fl}$.

A Gating scheme explaining the identification of the Ly6G^{-}, F4/80^{+}, CD11b^{+}, and NRP1^{+} mononuclear phagocytes in retinas and sclera-choroid-RPE cell complexes. 1. Gating of live cells, 2. Removal of doublets, 3. Selection of viable cells, 4. Exclusion of neutrophils, 5. Gating of mononuclear phagocytes, 6. Gating of NRP1^{+} mononuclear macrophages.

B FACS histogram of APC-conjugated rat IgG2A isotype control (red) versus anti-mNRP1 APC-conjugated rat IgG2A (R&D systems) (blue).

C FACS histogram of FMO (fluorescence minus one) versus anti-mNRP1 APC-conjugated rat IgG2A.

D, E Quantification of NRP1-positive microglia (Ly6G^{-}, F4/80^{+}, CD11b^{+}, CX3CR1^{hi}, CD45^{lo}, NRP1^{+}) in retinas and sclera-choroid-RPE cell complexes in Naïve (non-burned) mice (D); $n = 4$ and at D3 (E); $n = 4$.

F, G Quantification of mononuclear phagocytes (Ly6G^{-}, F4/80^{+}, CD11b^{+}) in retinas and sclera-choroid-RPE cell complexes in Naïve (non-burned) mice (F); $n = 5$ (LysM-Cre/*Nrp1*$^{+/+}$), 4 (LysM-Cre/*Nrp1*$^{fl/fl}$) and at D3 (G); $n = 4$.

H Compilation of representative compressed Z-stack confocal images of FITC–dextran-labeled CNV and IB4-stained laser impact area from LysM-Cre/*Nrp1*$^{+/+}$ and LysM-Cre/*Nrp1*$^{fl/fl}$ mice at D7. Scale bar: 20 μm.

I–K Quantification of area of FITC–dextran-labeled CNV (I), isolectin B4 (IB4)-stained laser impact area (J) and the ratio of FITC/IB4 per laser burn (K) relative to LysM-Cre/*Nrp1*$^{+/+}$ at D7; $n = 14$ burns (LysM-Cre/*Nrp1*$^{+/+}$), $n = 20$ burns (LysM-Cre/*Nrp1*$^{fl/fl}$).

Data information: All comparisons between groups were analyzed using a Student's unpaired t-test; $^{**}P < 0.01$, $^{***}P < 0.001$; error bars represent mean ± SEM; exact P values listed in Table 2.

© 2021 The Authors

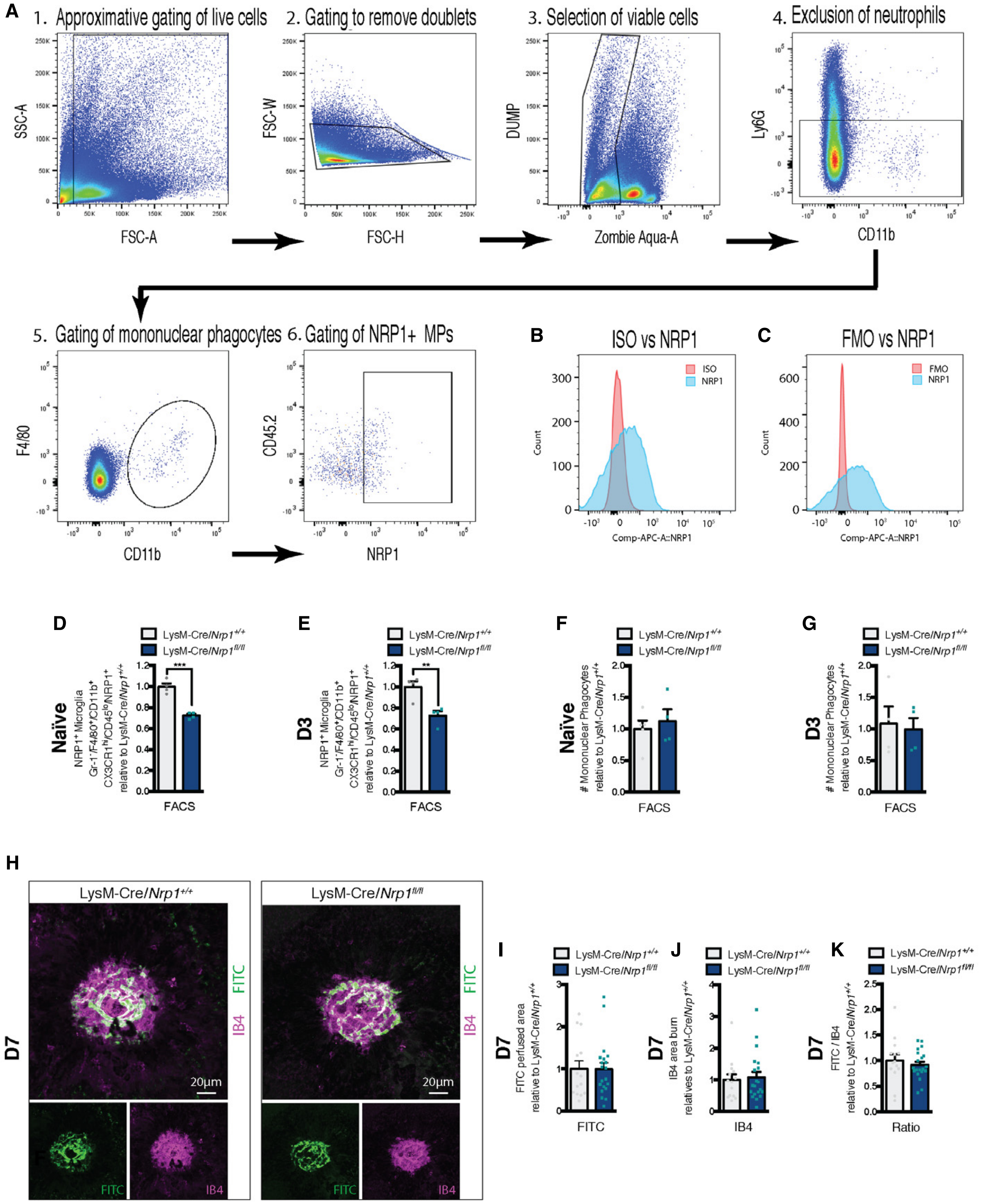
A
1. Approximative gating of live cells
2. Gating to remove doublets
3. Selection of viable cells
4. Exclusion of neutrophils
SSC-A
FSC-A
FSC-W
FSC-H
DUMP
Zombie Aqua-A
Ly6G
CD11b
5. Gating of mononuclear phagocytes
6. Gating of NRP1+ MPs
F4/80
CD45.2
NRP1
B
ISO vs NRP1
ISO
NRP1
Count
Comp-APC-A::NRP1
C
FMO vs NRP1
FMO
D
E
F
G
LysM-Cre/Nrp1+/+
LysM-Cre/Nrp1fl/fl
Naïve
D3
FACS
Mononuclear Phagocytes relative to LysM-Cre/Nrp1+/+
H
D7
FITC
IB4
20µm
I
J
K
FITC perfused area relative to LysM-Cre/Nrp1+/+
IB4 area burn relatives to LysM-Cre/Nrp1+/+
FITC / IB4 relative to LysM-Cre/Nrp1+/+
Ratio

Figure EV1.

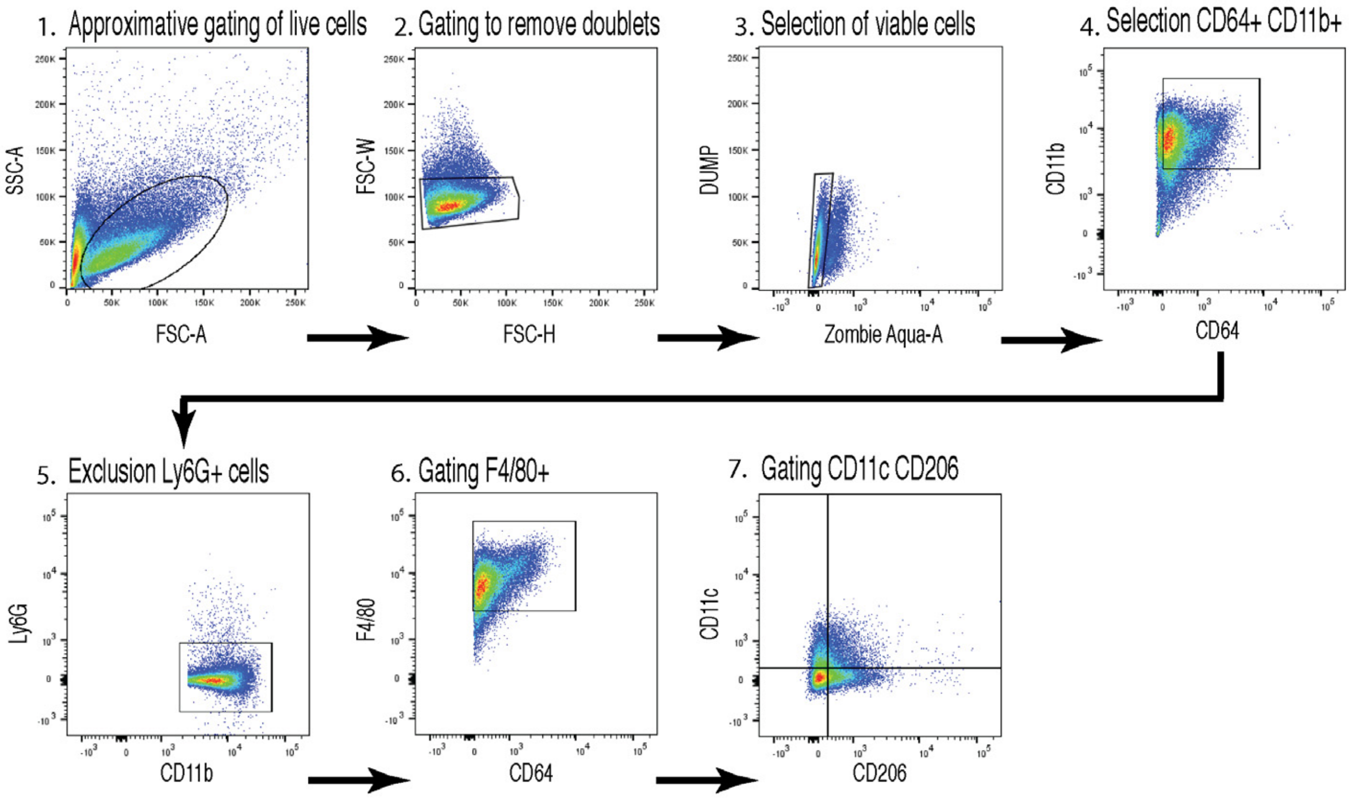

Figure EV2. Gating scheme explaining the identification of the Ly6G$^-$, F4/80$^+$, CD11b$^+$, CD11c$^+$, CD206$^-$ and Ly6G$^-$, F4/80$^+$, CD11b$^+$, CD11c$^-$, CD206$^+$ BMDMs.

1. gating of live cells, 2. removal of doublets, 3. selection of viable cells, 4. Selection of myeloid cells (CD64+) 5. exclusion of neutrophils, 6. gating of mononuclear macrophages, 7. Gating on CD11c and CD206.

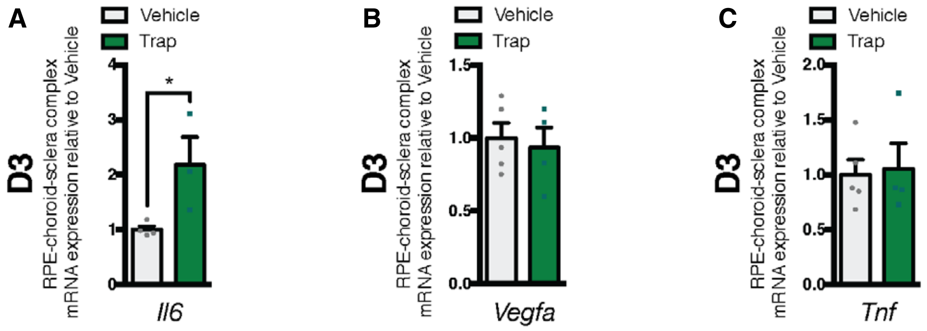

Figure EV3. Inflammatory state of RPE-choroid-sclera complexes following treatment with trap.

A–C mRNA expression of inflammation markers relative to vehicle in mouse RPE-choroid-sclera complexes at D3 for *Il6* (A); $n = 4$ (vehicle), $n = 4$ (trap), *Vegfa* (B); $n = 5$ (vehicle), $n = 4$ (trap), *Tnf* (C); $n = 5$ (vehicle), $n = 4$ (trap).

Data information: All comparisons between groups were analyzed using a Student's unpaired t-test; $*P < 0.05$; error bars represent mean ± SEM; exact P values listed in Table 2.
